# Supplementary material for: A stochastic assembly model for Nipah virus revealed by super-resolution microscopy
Source: Nat Commun. 2018 Aug 3;9:3050. doi: 10.1038/s41467-018-05480-2 (PMC6076310; doi:10.1038/s41467-018-05480-2)
Supplement: Supplementary file 1 — Supplementary Information [file 41467_2018_5480_MOESM1_ESM.docx]

**
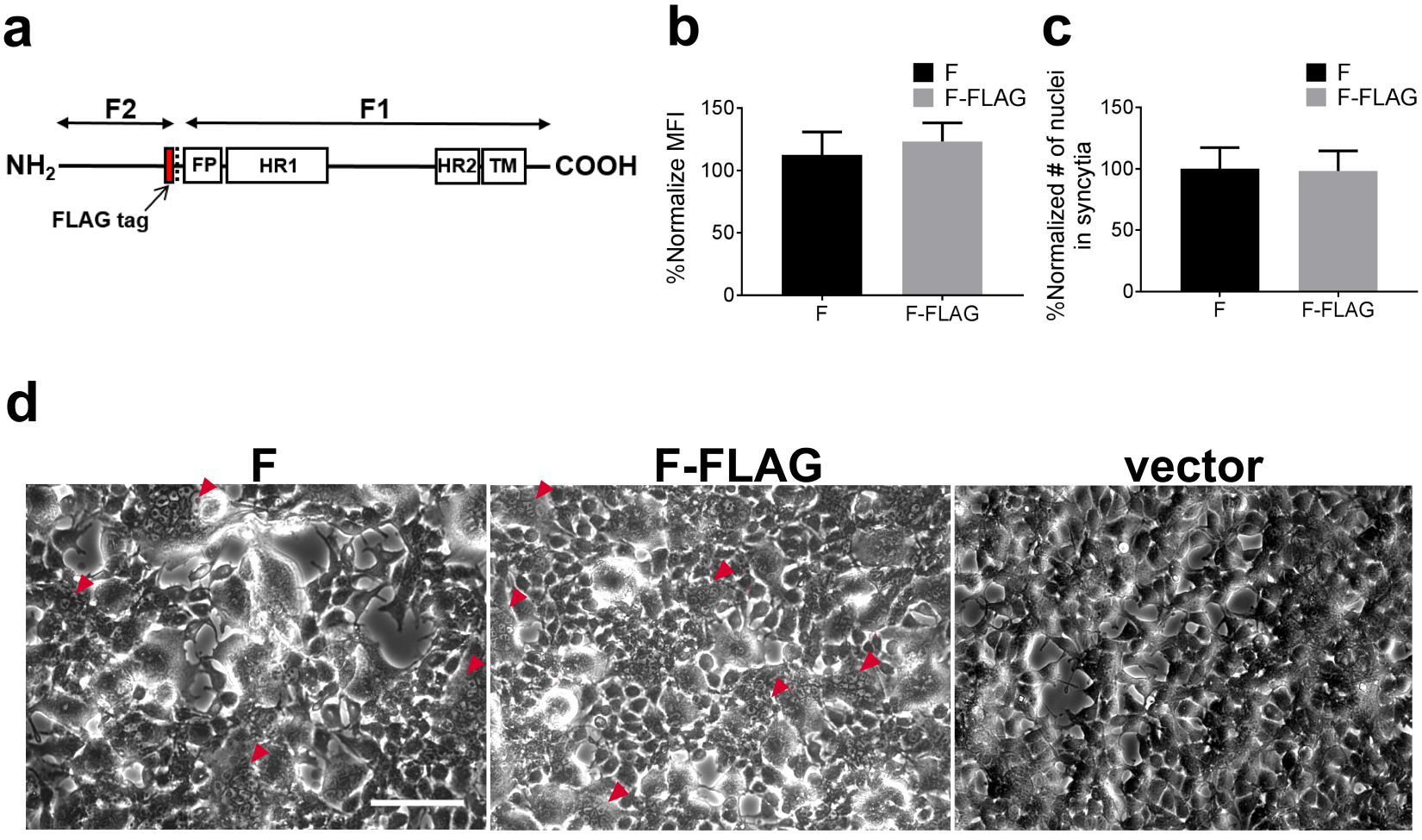
**

**Supplementary Figure 1: The FLAG-tagged NiV-F construct can induce cell-cell fusion. a,** diagram of the FLAG-tagged NiV-F (F-FLAG) construct used in the current study. NiV-F is a class I fusion protein which is cleaved into F1 and F2 subunits in the host cell. The FLAG tag (red box) is located at the C-terminus of the F2 subunit. **b**, Expression plasmids of the untagged F (F), F-FLAG were transfected in 293T cells. At 24 hrs post-transfection, the F constructs were probed using rabbit polyclonal anti-NiV-F antisera and Alexa Fluor 647 conjugated goat-anti-rabbit antibodies. The cell surface expression levels of the F constructs were determined by the median fluorescence intensity (MFI) at the 647 channel by flow cytometry. The MFI values were normalized to that of the F. **c and d,** the expression plasmids of HA-tagged G and F, F-FLAG were co-transfected into 293T cells. At 20 hrs post transfection, cell-cell fusion was observed using a phase contrast microscope at 20x magnification. **c**, the levels of cell-cell fusion were determined by counting the number of nuclei in syncytia (≥4 nuclei in a common membrane) in each field. The number of nuclei in syncytia was normalized to that of the F. **d,** Representative images of syncytia in cells expressing HA-tagged G and F, F-FLAG or empty vector. Similar results were observed in HeLa and Vero cells. Three independent experiments were performed.

**
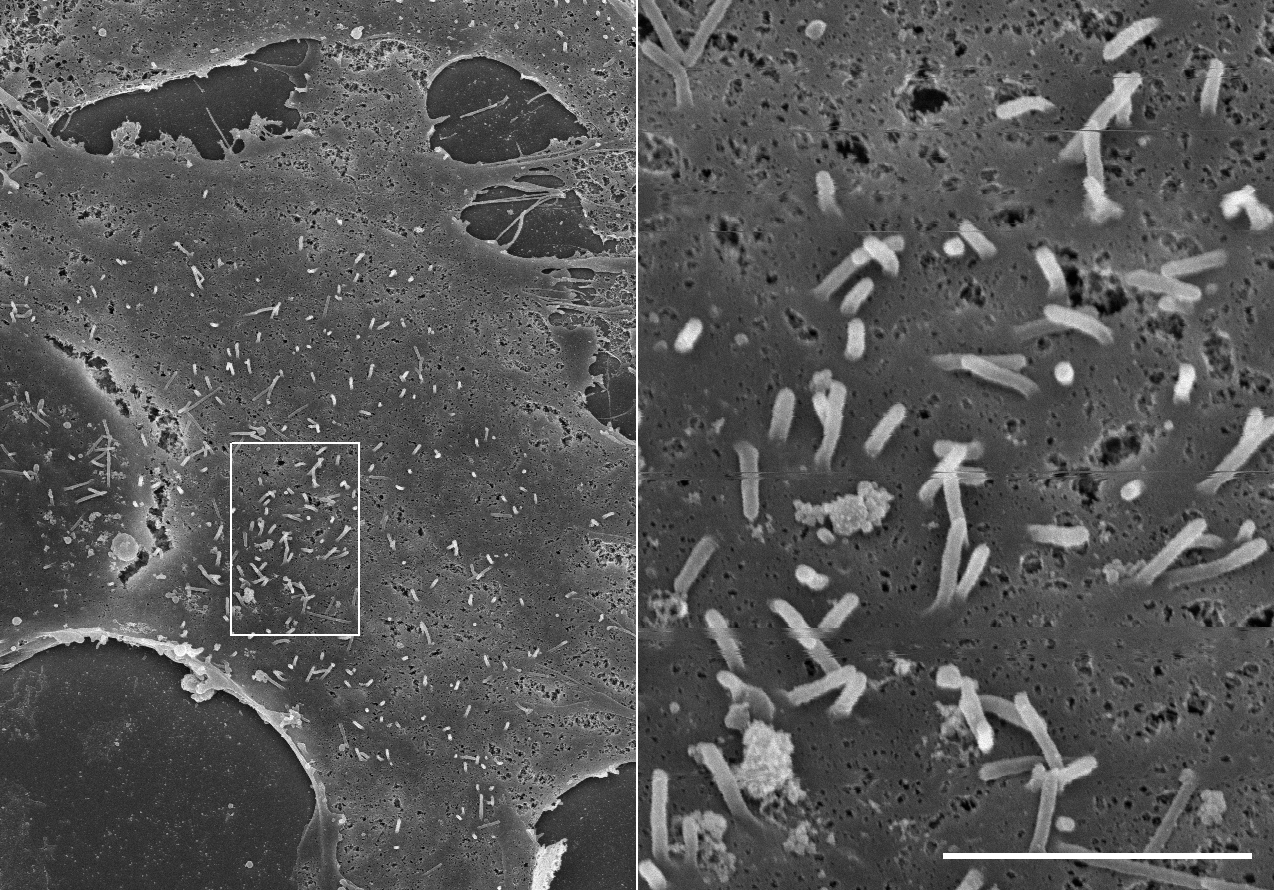
**

**Supplementary Figure 2: Protrusions on PK13 cell surface.** PK13 cells were seeded on fibronectin-coated glass coverslips, fixed and subjected to SEM imaging. One cell image out of three independent experiments (n=10) is shown. Scale bar: 10 um.


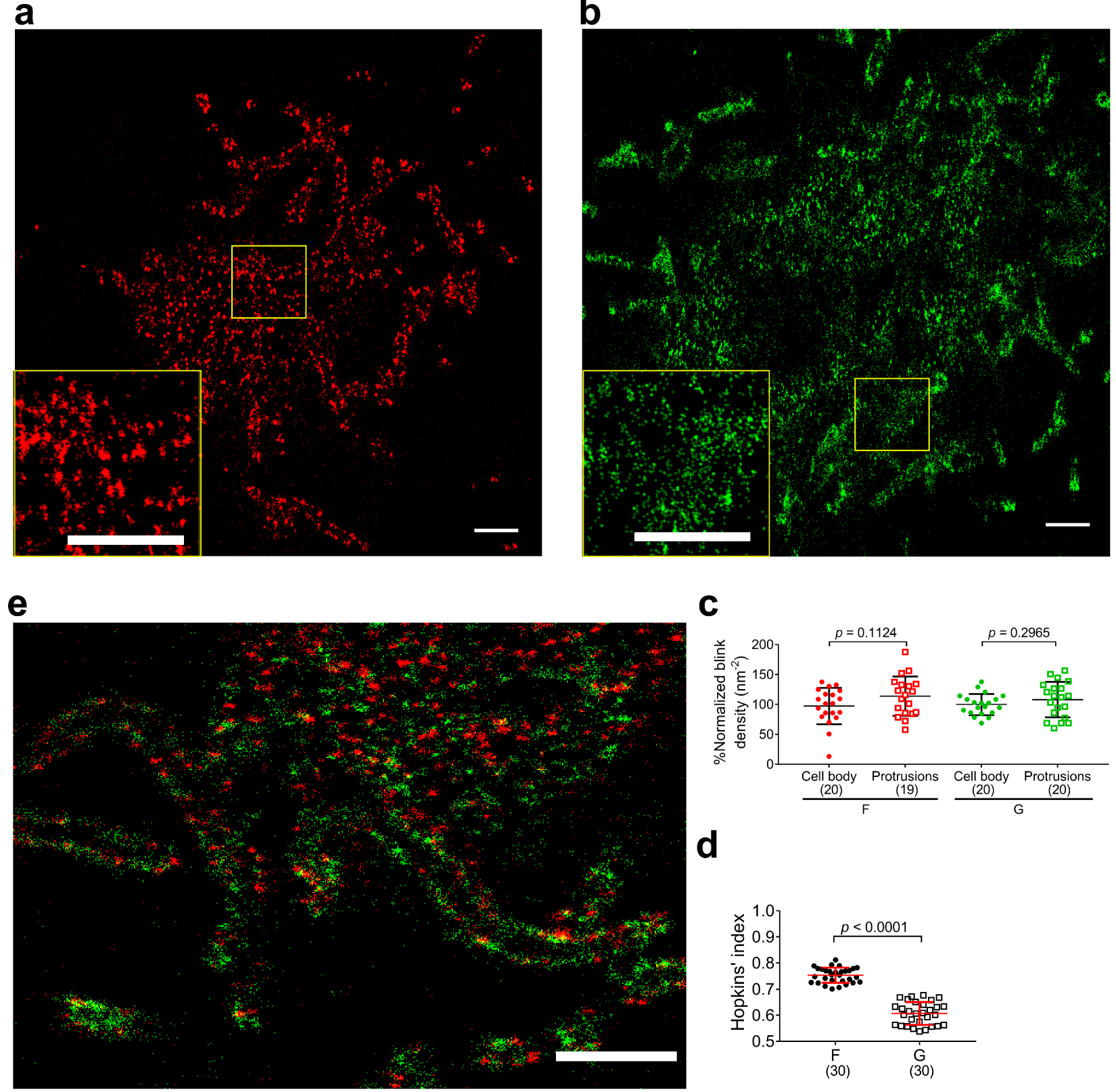


**Supplementary Figure 3: Distribution of NiV envelope glycoprotein is independent on the expression levels on the plasma membrane of PK13 cells.** PK13 cells expressing NiV-F or/and G were fixed at 16 hrs post-transfection and immunostained. Without permeabilization, NiV-F was immunostained by using a mouse anti-FLAG primary antibody and G a rabbit anti-HA primary antibody. For single color SMLM, Alexa Fluor 647 secondary antibodies were used for detection. For dual color SMLM, Alexa Fluor 647 secondary antibodies were used for detection of F, and Cy3B secondary antibodies for G. **a, b,** *x-y* cross section (100 nm thick in *z*) at the dorsal surface (position 3 in Fig. 1a) of a representative cell expressing F (**a**), or G (**b**). The boxed region is enlarged to show the detailed distribution pattern. **c**, Comparison analyses of the localization densities of the F (red) or G (green) at the cell body versus membrane protrusions. Each data point was calculated using an area of 0.2 x 0.2 um^2^. All data were normalized to the mean of the cell body. **d,** Hopkins’ index of the F and G localizations. Lines represent the mean value and SD. The sample size is indicated in the parentheses. The *p* values were determined by two-tailed, unpaired *t*-test with Welch correction. **e,** *x-y* cross section (100 nm thick in *z*) of a region at the dorsal surface (position 3 in Fig. 1a) of a representative cell co-expressing F (red) and G (green) with a pixel size of 10 nm. Scale bars: 1 um. One representative cell image out of three independent experiments (n≥30) is shown.

**
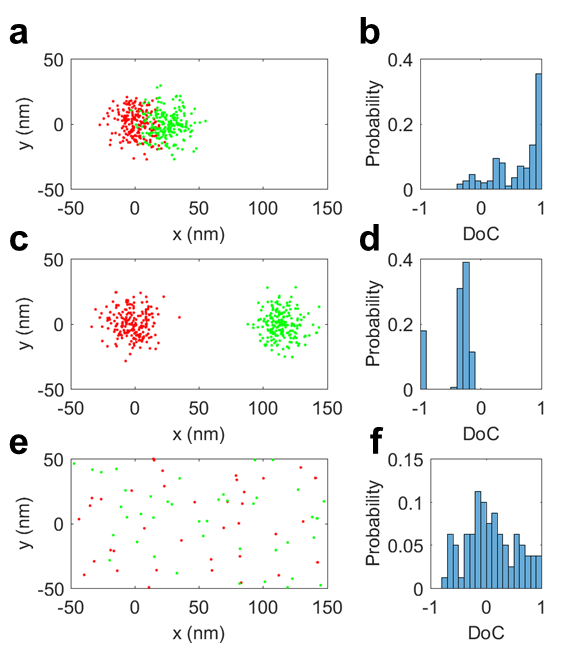
**

**Supplementary Figure 4: Two-color simulations of CBC analysis with R_max_ = 100 nm.** Two overlapped clusters (red and green) (**a**) produce DoC values mostly in the positive region (**b**). Two clusters with a distance larger than 100 nm (**c**) produce DoC values in the negative range (**d**). Randomly distributed points (**e**) give DoC values centered near zero (**f**).


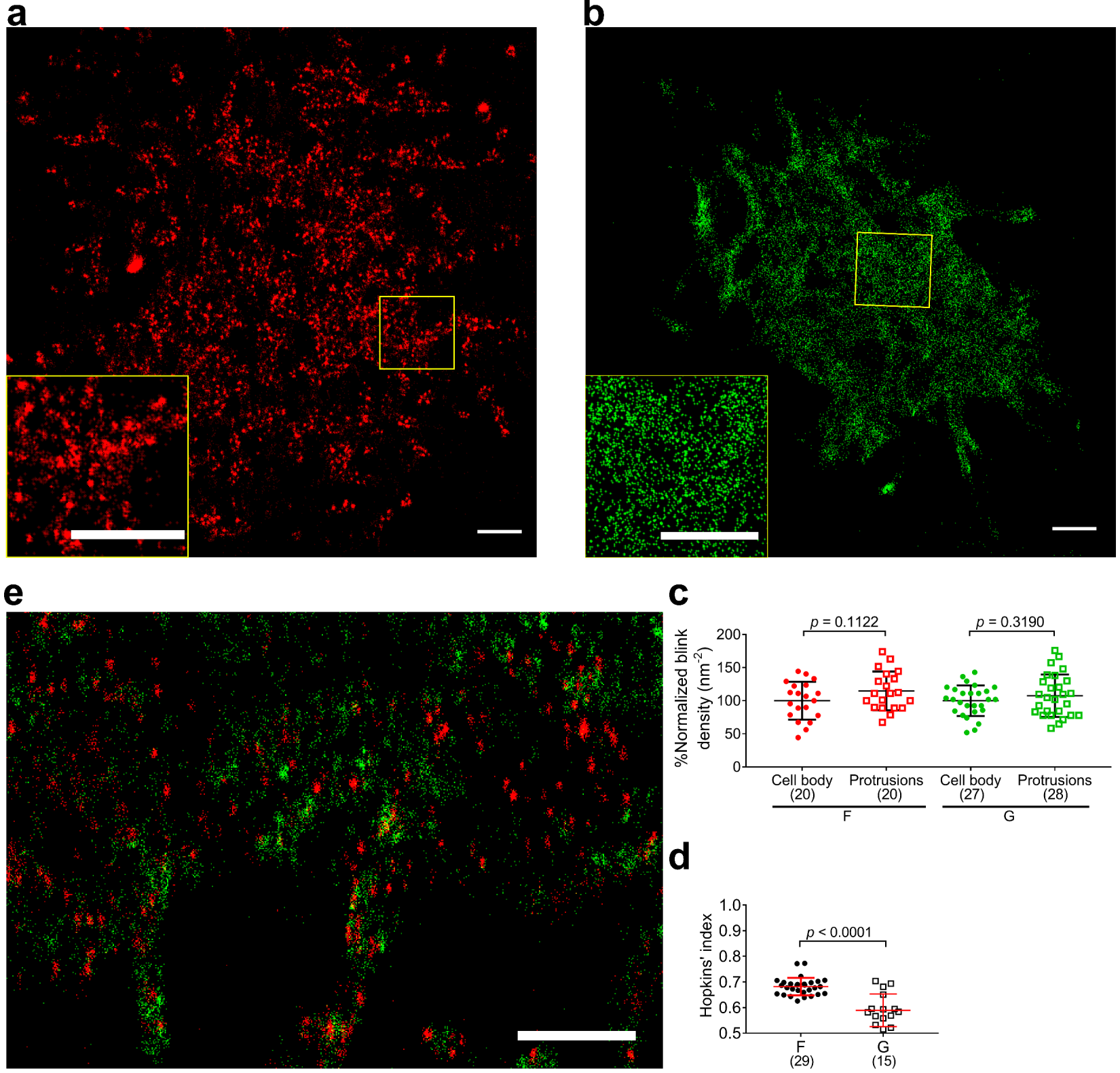


**Supplementary Figure 5: Distribution of NiV envelope glycoproteins is independent of the presence of ephrin-B2 and -B3 receptors in HeLa cells.** HeLa cells expressing NiV-F or/and G were fixed at 24 hrs post-transfection and immunostained. Without permeabilization, NiV-F was immunostained by using a mouse anti-FLAG primary antibody and G a rabbit anti-HA primary antibody. For single color SMLM, Alexa Fluor 647 secondary antibodies were used for detection. For dual color SMLM, Alexa Fluor 647 secondary antibodies were used for detection of F, and Cy3B secondary antibodies for G. **a, b,** *x-y* cross section (100 nm thick in *z*) at the dorsal surface (position 3 in Fig. 1a) of a representative cell expressing F (**a**), or G (**b**). The boxed region is enlarged to show the detailed distribution pattern. **c**, Comparison analyses of the localization densities of the F (red) or G (green) at the cell body versus membrane protrusions. Each data point was calculated using an area of 0.2 x 0.2 um^2^. All data were normalized to the mean of the cell body. **d,** Hopkins’ index of the F and G localizations. Lines represent the mean value and SD. The sample size is indicated in the parentheses. The *p* values were determined by two-tailed, unpaired *t*-test with Welch correction. **e,** *x-y* cross section (100 nm thick in *z*) of a region at the dorsal surface (position 3 in Fig. 1a) of a representative cell co-expressing F (red) and G (green) with a pixel size of 10 nm. Scale bars: 1 um. One representative cell image out of three independent experiments (n≥30) is shown.


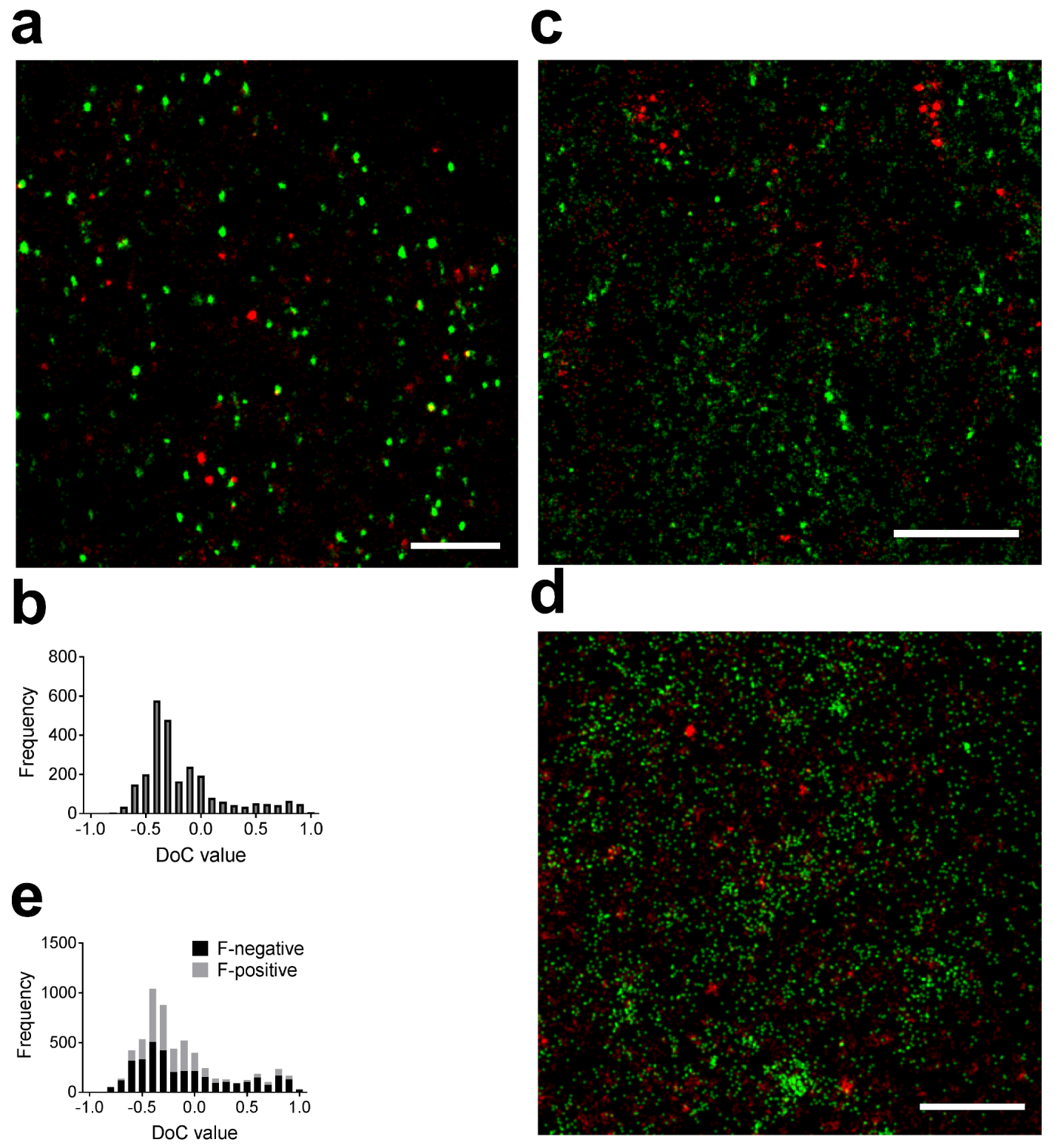


**Supplementary Figure 6: F or G do not significanly co-localize with M at the plasma membrane.** In **a and c,** PK13 cells expressing M and NiV-F or NiV-G were fixed at 24 hrs post-transfection and immunostained. After permeabilization, NiV-F was immunostained by using a mouse anti-FLAG primary antibody, G a rabbit anti-HA primary antibody and M a goat anti-GFP primary antibody. For dual color SMLM, Alexa Fluor 647 secondary antibodies were used for detection of M, and Cy3B secondary antibodies for detection of G or F. **a,** *x-y* cross section (100 nm thick in *z*) of the dorsal surface (position 3 in Fig. 1a) of a representative cell expressing F (green) and M (red). **b,** Distribution of the DoC values between F and M. **c,** *x-y* cross section (100 nm thick in *z*) of the dorsal surface (position 3 in Fig. 1a) of a representative cell expressing G (green) and M (red). **d,** *x-y* cross section (100 nm thick in *z*) of the dorsal surface (position 3 in Fig. 1a) of a representative cell expressing F, G (green) and M (red). PK13 cells expressing M, F and G were fixed at 24 hrs post-transfection, permeabilized and immunostained. An Alexa 488 secondary antibody was used to detect the expression of F. M and G were detected by SMLM as previously described. The signal of F was determined after the GFP signal from M was photobleached. The F-positive cells were selected for SMLM imaging of the G and M. **e,** Distribution of the DoC values between G and M in the absence (black) and presence (gray) of F. One representative cell image out of three independent experiments (n≥20) is shown.
